# Treatment Considerations for Obstructive Sleep Apnea in Pediatric Down Syndrome

**DOI:** 10.3390/children8111074

**Published:** 2021-11-22

**Authors:** Erica Gastelum, Marcus Cummins, Amitoj Singh, Michael Montoya, Gino Luis Urbano, Mary Anne Tablizo

**Affiliations:** 1 School of Medicine, University of California San Francisco, Fresno, CA 94143, USA; marcus.cummins@ucsf.edu (M.C.); amitoj.singh@ucsf.edu (A.S.); 2 Department of Pediatrics, Undergraduate Medical Education, University of California San Francisco, Fresno, CA 93721, USA; mimontoya@ucdavis.edu; 3 School of Medicine, University of California Davis, Sacramento, Fresno, CA 95817, USA; 4 School of Medicine and Public Health, Ateneo de Manila University, Pasig 1604, Philippines; gino.urbano@obf.ateneo.edu; 5 Department of Pulmonology, Valley Children’s Hospital, Madera, CA 93720, USA; mtablizomd@gmail.com; 6 Department of Pulmonology, Stanford Children’s Health, Lucille Packard Children’s Hospital, Palo Alto, CA 94304, USA

**Keywords:** obstructive sleep apnea, Down syndrome, trisomy 21, pediatric, adenotonsillectomy, continuous positive airway pressure

## Abstract

Children with Down syndrome (DS) are at high risk for developing obstructive sleep apnea (OSA) compared to children without DS. The negative impact of OSA on health, behavior, and cognitive development in children with DS highlights the importance of timely and effective treatment. Due to the higher prevalence of craniofacial and airway abnormalities, obesity, and hypotonia in patients with DS, residual OSA can still occur after exhausting first-line options. While treatment commonly includes adenotonsillectomy (AT) and continuous positive airway pressure (CPAP) therapy, additional therapy such as medical management and/or adjuvant surgical procedures need to be considered in refractory OSA. Given the significant comorbidities secondary to untreated OSA in children with DS, such as cardiovascular and neurobehavioral consequences, more robust randomized trials in this patient population are needed to produce treatment guidelines separate from those for the general pediatric population of otherwise healthy children with OSA. Further studies are also needed to look at desensitization and optimization of CPAP use in patients with DS and OSA.

## 1. Introduction

Down Syndrome (DS) or trisomy 21, is the most common chromosomal disorder, accounting for about 1 in every 700 births in the United States each year [1]. It is associated with delayed psychomotor development, hypotonia, and characteristic dysmorphic features including brachycephaly, flat face, flat nasal bridge, mandibular and maxillary hypoplasia, and narrow nasopharynx [2]. Common medical comorbidities include airway abnormalities such as laryngomalacia and tracheomalacia, congenital heart defects, hearing disorders, vision disorders, congenital defects of the gastrointestinal tract, celiac disease, hypothyroidism, obesity, and obstructive sleep apnea [2].

Obstructive sleep apnea (OSA) is defined as recurrent episodes of complete or partial upper airway obstruction which can result in abnormalities in oxygenation and, ventilation and disruption of sleep continuity. OSA occurs at a significantly higher rate in children with DS than in those without [3]. The prevalence of OSA in DS varies based on the population studied. A large cohort study reported a 66.4% prevalence of OSA in DS, and even in those without a history of classic symptoms such as snoring or witnessed apnea the prevalence was 53.8% [4]. A 2018 meta-analysis, which included 1200 children with DS, reported a 76% prevalence of OSA based on an apnea–hypopnea index (AHI) of >1.5 events/h [5]. This is compared to a 1–4% prevalence of OSA in the general pediatric population [6]. 

The pathophysiology of OSA in children with DS is multifactorial. Predisposing factors include craniofacial abnormalities (mandibular or maxillary hypoplasia), airway abnormalities (narrow nasopharynx, laryngomalacia, tracheomalacia), hypotonia, and obesity [5,7,8]. This pattern of craniofacial hypoplasia is associated with a significant reduction in the cross-sectional area of the upper airway in mouse models of DS [9]. In addition to this reduction in volume, children with DS have enlarged structures within the airway, such as adenotonsillar hypertrophy and macroglossia [10]. Generalized hypotonia is nearly universal in children with DS [11]. The neuroanatomic localization for hypotonia appears to be in the cerebellum—one of many structures that experience disrupted neurogenesis during fetal development in children with DS [12]. Poor tone within the airway predisposes these children to glossoptosis, laryngomalacia, and hypopharyngeal collapse [10,13]. 

The combination of reduced airway volume, enlarged tonsils and adenoids, macroglossia, and hypotonia is a setup for recurrent upper airway obstruction during sleep This is further exacerbated by children with DS’s predisposition toward obesity—a major risk factor for the development and progression of OSA in the general population [14]. A nationwide sample of Dutch children revealed that children with DS were more likely to be overweight (25.5% vs. 13.3% in boys; 32.0% vs. 14.9% in girls) and obese (4.2% vs. 1.8% in boys; 5.1% vs. 2.2% in girls) compared to children without DS. These rates were observed with or without concomitant medical disorders [15]. Factors that increase the likelihood of obesity in children with DS include increased leptin, decreased resting energy expenditure, and unfavorable diets [16]. In addition, subclinical hypothyroidism is common in children with DS, which can also lead to obesity. In a cohort of DS children with hypothyroidism, initiation of L-thyroxine resulted in a reduction in body mass index [17]. Furthermore, musculoskeletal complications, such as pes planus (which occurred in 91% of a cohort of 503 DS children), inflammatory arthritis, and scoliosis, are common in children with DS [18]. These conditions, in combination with hypotonia, may make ambulation and exercise difficult and contribute to decreased levels of physical fitness and activity observed in children and adolescents with DS [19]. 

Untreated or poorly treated OSA has been found to have a negative impact on behavior and development in children. In a cross-sectional study of 53 children with DS, 51 (96%) were reported to have OSA. In these children, negative behaviors were positively correlated with AHI and developmental quotient was negatively correlated with AHI [20]. Furthermore, studies have shown that children with DS and OSA exhibit decreased language function and impaired working memory, emotional control, and executive function compared to those without OSA [21,22,23]. In addition, OSA is associated with an increased risk for several medical conditions, especially cardiovascular comorbidities. These include, coronary artery disease, arrhythmias, congestive heart failure, pulmonary hypertension, and cerebrovascular accidents [24]. Increased prevalence of left-to-right cardiac blood flow and higher baseline pulmonary resistance in children with DS further increases their risk of pulmonary hypertension [25]. The negative impact of OSA on health, behavior, and cognitive development in children with DS highlights the importance of timely and effective treatment. Unfortunately, the presence of several predisposing factors discussed above makes successful treatment extremely difficult. In this report, we aim to review the available treatment options for OSA, discuss the challenges and reasons for failure, and identify emerging therapeutic strategies that may be beneficial for children with DS.

## 2. Methods

A PubMed, EBSCO, and Google Scholar database search from January 2010 up to October 2021 was conducted using the terms: obstructive sleep apnea, Down’s syndrome, Downs syndrome, trisomy 21, pediatric, tonsillectomy, positive airway pressure, and treatment options. Cited references in selected articles were reviewed as well. Four independent reviewers reviewed eligible articles through titles and abstracts, and full articles were analyzed for eligibility and data abstraction. 

**Figure 1:**
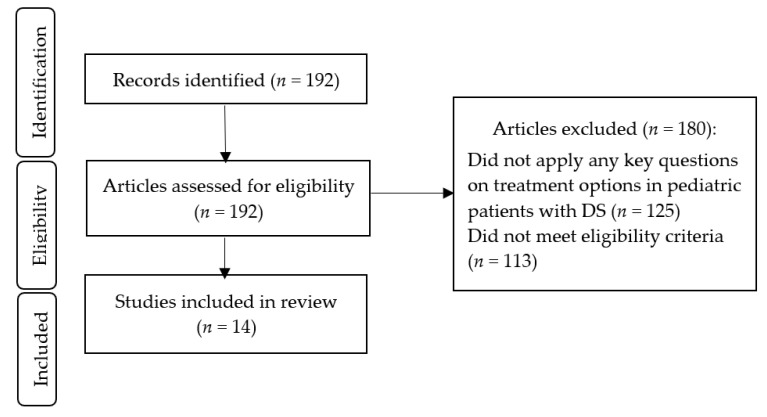
Flow chart of study selection. DS: Down Syndrome.

The eligibility criteria for including studies in the present review were as follows: (a) studies that included children and adolescents with DS, aged 0–19 years; (b) original research with a prospective or retrospective cohort, cross-sectional, longitudinal, case–control, or randomized controlled trial design administered in the hospital, outpatient clinic, community setting, or school; (c) studies that looked into treatment strategies such as adenotonsillectomy, lingual tonsillectomy, positive airway pressure therapy, high-flow nasal cannula, pharmacologic therapy, myofunctional therapy, hypoglossal nerve stimulation, drug-induced sleep endoscopy, or weight loss/weight reduction. We did not exclude studies that targeted children with DS who also had other medical conditions or comorbidities such as obesity, hypothyroidism, cardiovascular disease, or diabetes. We did not exclude studies based on sample size. Articles that were excluded were case reports, meta-analyses, reviews, and non-English language studies. The search identified 192 results. Exclusion of studies occurred in two phases: (1) records that did not apply any key questions on treatment options in pediatric patients with DS (*n* = 125) and (2) articles that did not meet eligibility criteria (*n* = 113). After eligibility screening, 14 studies were included. Figure 1 illustrates the study selection process for this present review.

## 3. Diagnosis

Attended in-laboratory nocturnal polysomnography (PSG) is the gold standard for the diagnosis of OSA in children with DS [25]. Confirmatory presence of OSA on PSG determines its severity including abnormalities in oxygenation and ventilation, which will help in preoperative planning. Given that parent reports and PSG results do not always correlate, the American Academy of Pediatrics (AAP) recommends a PSG referral for all children with DS by 4 years of age [26]. However, PSG is not always available or accessible. A large, 5-year, single-center, retrospective cohort study included more than 700 children with DS and compared rates of OSA screening before 4 years of age both before and after the release of the 2011 AAP guidelines. They found no significant difference in screening rates pre- and post-guidelines (63.4% vs. 59.4%, respectively; *p* = 0.26) [27]. A follow-up study including the 460 children born post-guidelines investigated the demographic and clinical characteristics associated with adherence to OSA screening. There were no significant differences based on sex, insurance status, or socioeconomic status among children who did or did not obtain a screening PSG. However, children who lived further from the medical center were less likely to obtain screening, and among children with medical comorbidities, those with hypothyroidism and pulmonary aspiration were more likely to obtain a screening PSG [28]. 

Due to the considerable proportion of children with DS that remain unscreened, studies have evaluated the utility of alternative screening and diagnostic methods. Home sleep test (HST) is commonly used in the diagnosis of OSA in adults but has not been well studied in children. Although HST can miss a diagnosis and underestimate severity of OSA due to the absence of electroencephalography and carbon dioxide monitoring, it can be considered as an alternative diagnostic procedure for children with DS who cannot tolerate in-laboratory PSG. A multicenter study investigated the use of home pulse oximetry (HPO) to identify children with DS at increased risk for OSA and reported a sensitivity for moderate-to-severe OSA up to 96% depending on the HPO parameters used [29]. The use of home polygraphy has also garnered interest, and one study in children with DS reported a 100% sensitivity and 83% specificity when AHI ≥ 3 was used for the diagnosis of OSA [30]. This will likely miss mild OSA, and it has not yet been determined at what threshold OSA results in sequalae.

Children with DS are predisposed to persistent OSA due to airway obstruction at multiple sites. Drug-induced sedation endoscopy (DISE) is increasingly being used to evaluate the level of airway obstruction prior to surgical intervention and to guide targeted treatment. DISE allows direct visualization of the upper airway at multiple levels and can help classify the level, type, and severity of obstruction [31]. In 25 children with DS who underwent DISE-directed surgery and had pre- and post-operative PSG data, there was a statistically significant reduction in the obstructive apnea–hypopnea index (oAHI) from 11.4/h to 5.5/h [31]. However, in a different population of 24 children with DS who obtained PSGs before and after DISE-directed surgery, oxygen nadir was the only PSG parameter that demonstrated a statistically significant improvement [32]. Additional data among larger cohorts will be required to determine whether DISE-directed treatment improves outcomes in children with DS.

Cine magnetic resonance imaging (MRI) is another tool used to identify the level of obstruction by three-dimensional analysis of the upper airway in patients with DS who had previously undergone AT but have refractory OSA. This procedure is done under sedation. In one study with 27 patients with DS and refractory OSA, the cine MRI was able to identify multiple sites of airway obstruction including glossoptosis (63%), recurrent and enlarged adenoid tissue (63%), enlarged lingual tonsils (30%) and hypopharyngeal collapse (22%) [10]. 

## 4. Treatment Options

As pediatric patients with trisomy 21 are more susceptible to the cardiovascular and neurocognitive consequences of OSA, early diagnosis and treatment is critical [25]. First-line treatment options for these patients generally include adenotonsillectomy (AT) and subsequently continuous positive airway pressure (CPAP) therapy for those with residual OSA. Adjunctive therapies are being increasingly considered, as many of these patients with DS have residual OSA post-surgery and often do not tolerate CPAP therapy well. These options include performing lingual tonsillectomy, use of high flow nasal cannula (HFNC), medical management with anti-inflammatory medications and myofunctional therapy. A newer therapy called hypoglossal nerve stimulation is now being used for refractory OSA in patients with DS [25]. The literature on these additional options is still lacking. A summary of the available studies for treatment options of OSA in pediatric patients with DS is shown in Table 1.

### 4.1. Adenotonsillectomy

AT is a common surgical procedure for pediatric patients with OSA, and it is widely used as first-line treatment for patients with adenoid and tonsillar hypertrophy [25]. According to the AAP and the American Academy of Otolaryngology Head and Neck Surgery, it has an approximately 80% success rate in treating otherwise healthy children with OSA [46]. In children with DS, there are only a few studies, and these often have small sample sizes.

A retrospective study by Nerfeldt and Sundelin examined 14 pediatric trisomy 21 patients with OSA before and after AT. Preoperative PSG showed a median AHI of 12.4 and a postoperative AHI of 11.4 one year after surgery (*p* = 0.054) [34]. Therefore, indicating minimal reduction of OSA severity. They also examined OSA-18, a validated quality-of-life measurement that is scored on a scale of 18 to 126 for healthy children with OSA who undergo adenotonsillectomy. Higher scores on this questionnaire indicate greater OSA severity, with 31.2 ± 10.4 representing the mean score of patients with no OSA symptoms, and >60 representing moderate impact on quality of life [47]. The preoperative median OSA-18 score in this group was 53.5, compared to 36 at one year after surgery (*p* = 0.032) [34]. Although there were improvements in children with DS using OSA-18 in this study, it is difficult to know how much of this will apply to the larger population of children with DS.

A retrospective chart review by Ingram et al. examined 75 children with DS who underwent tonsillectomy between 2009 and 2015 and had concurrent adenoidectomy or previous adenoidectomy. AHI at baseline was 21.3 ± 19.7, and after six months, it had decreased to 8.0 ± 8.1 [41]. A cohort study by Sudarsan et al. looked at 37 pediatric patients with either DS or mucopolysaccharidosis (MPS) and the efficacy of AT in this subgroup. They found a decrease in AHI from a baseline of 3.83 ± 1.36 to 2.62 ± 0.87 at six months indicating that surgery reduces the number of apneas and hypopneas during sleep [43]. However, as the patients in this study had a low baseline AHI, it is difficult to determine the significance of the clinical benefit of AT. This further emphasizes the need for more data in this specific subgroup.

Another study by Shete et al. compared the efficacy of AT in children with DS to those without DS. This was assessed by AHI, rapid eye movement (REM)-AHI, lowest oxygen saturation, and sleep disruption. In all, 11 patients with DS and 9 patients without DS were included in the study. The average total AHI and REM-AHI for the children with DS on pre-operative PSG was 15.3 and 30.5 respectively; compared to 21.1 and 32.5 for the children without DS [45]. After AT, the total AHI and REM-AHI for the children with DS decreased to 9.1 and 21.9, respectively. For the children without DS, total AHI and REM-AHI decreased to 1.8 and 6.03, respectively. The lowest oxygen saturation before AT was around 70% for both groups. Though there was no change for the group with DS, the oxygen saturation increased to an average of 89.6% for the children without DS [45]. For both cohorts of children, AT did not significantly affect distribution of time in different stages of sleep, the sleep efficiency (ratio of total sleep time to time in bed), and the mean arousal index (number of arousals per hour of total sleep time). For the group of children with DS who underwent AT, 55% required CPAP or BIPAP, and 18% required nocturnal oxygen. On the other hand, in this study, none of the children without DS required further treatment after AT [45].

In summary, a few studies have shown an improvement in OSA after AT for pediatric patients with trisomy 21. However, the cure rate for the general pediatric population has not been reproduced in this specific subgroup, and over 50% of patients with DS will have clinically significant OSA post-operatively [45]. Many patients with residual disease will need other treatment options to treat OSA.

### 4.2. Lingual Tonsillectomy

Lingual tonsil hypertrophy can cause persistent airway obstruction in patients with DS who have gone through AT. Few studies have shown lingual tonsillectomy (LT) as an effective adjuvant surgical treatment for DS patients with persistent airway obstruction related to obstructive lingual tonsil hypertrophy following adenoidectomy and palatine tonsillectomy [40]. Prosser et al. examined the efficacy of lingual tonsillectomy for pediatric DS patients post-AT. 

Overall, 21 patients met the inclusion criteria for the study, and the mean age was 9.3 ± 4.3 years. Patients underwent lingual tonsillectomy at a tertiary care center between 2003 and 2013 and had polysomnography before and after the procedure [40]. The median AHI before the surgery was 9.1 events/h compared to 3.7 events/h after the surgery (*p* < 0.0001). The mean oxygen saturation nadir prior to surgery was 84% compared to 89% after the surgery (*p* = 0.004) [40].

Therefore, this group of patients had reduced OSA severity after lingual tonsillectomy. Patients with DS who have persistent OSA after AT should be examined to determine eligibility for lingual tonsil hypertrophy [40].

### 4.3. CPAP Therapy

CPAP is one of the more common therapies used to treat pediatric DS patients with OSA if they fail surgery or if they are not surgical candidates. This non-invasive intervention assists in preventing airway collapse at night by applying positive pressure [40]. Many studies have shown the efficacy of this therapy in terms of sleep quality and OSA-related comorbidities for adult patients with strong adherence to treatment. In patients with excellent compliance, CPAP therapy has also been shown to be efficacious in children; however, studies are still lacking to examine the efficacy of this treatment in pediatric patients with trisomy 21 [25,48]. 

Sudarsan et al. looked at the efficacy of AT and CPAP therapy for pediatric patients with DS or MPS with comorbid OSA. They found that 36 patients in the CPAP group completed the study and reported nightly CPAP use [43]. Baseline AHI dropped from 3.46 ± 1.67 to 1.09 ± 0.61 at 6 months (*p* = 0.001) showing that CPAP therapy was effective at reducing the severity of OSA in patients with DS who regularly used their machine [43]. The low baseline AHI indicates the need for more data on pediatric DS patients with greater OSA severity to determine just how efficacious CPAP therapy can be in this population. 

While regular usage of CPAP has been shown to reduce the severity of OSA in a few studies, adherence to treatment remains an issue [25,49]. A retrospective chart review by Chawla et al. examined the usage of CPAP by pediatric patients with DS and looked at 25 children who were started on CPAP with a median age at initiation of 8.4 years. Of those, 11 children had “poor” sustained usage, defined as less than four hours per day; 5 children out of these 11 ended up discontinuing the treatment. They noted that 3 children had “moderate” usage, defined as four to six hours per night, and 10 children had “good” usage, defined as greater than six hours per day [49].

### 4.4. High-Flow Nasal Cannula

High flow nasal cannula (HFNC) has been used in pediatric patients with DS when they have difficulty adhering to CPAP. Amaddeo et al. studied this therapy in eight patients, six of whom had DS. They found that only three of the six were successfully managed with HFNC [38]. More research is needed to further understand the efficacy of alternative methods such as HFNC in pediatric patients with DS, as well as the challenges in adherence for this specific population.

### 4.5. Hypoglossal Nerve Stimulation

For patients who failed conventional OSA treatment such as AT and failed CPAP use, hypoglossal nerve stimulation may be considered. The criteria for hypoglossal nerve stimulation for adolescents and young adults with DS include all of the following: age 10 to 21 years, an AHI >10 and <50 with less than 25% central apneas after AT, ineffective treatment with CPAP, a BMI ≤ 95th percentile for age, and non-concentric retropalatal obstruction confirmed on DISE [33,39,50]. This therapy involves implanting a device that delivers an electrical impulse to the anterior branches of the hypoglossal nerve in response to respiratory variation. This results in protrusion of the tongue base, which alleviates some of the upper airway obstruction [39]. 

Diercks et al. conducted a case series on the first six adolescents with DS and severe OSA who had persistent symptoms after AT and underwent hypoglossal nerve stimulator implantation. They looked at adherence to therapy, measured by hours of use recorded by the device, and efficacy of the intervention by looking at AHI and OSA-18 [39]. In the six patients, the therapy was well tolerated with an average use of 5.6 to 10 h per night. In terms of efficacy, patients demonstrated a 56% to 85% reduction in AHI at the six to 12 month follow up and an average decrease in score of 1.5 on the OSA-18 [39]. The researchers categorized a change in OSA-18 score of 1.5 or greater as large, 1.0 to 1.4 as moderate, 0.5 to 0.9 as mild, and less than 0.5 as trivial [39].

Another case series by Caloway et al. investigated the efficacy and tolerance of this therapy amongst 20 adolescents with DS. The participants completed a PSG at 2 months after the implantation, which showed a median reduction in AHI of 85%. The median nightly use for these children was approximately 9.21 h per night [33]. Hypoglossal nerve stimulation appears to be well tolerated and efficacious in terms of reducing OSA severity in adolescent patients with DS who have persistent residual disease after AT. This study shows promise in treating OSA in DS, but the long-term efficacy of this therapy is not known. 

### 4.6. Tongue Based Procedure

Airway obstruction at the level of the base of the tongue is known to cause residual OSA after AT especially in patients with DS. The airway obstruction from the tongue may be related to the base of the tongue collapse especialy in patients with macroglossia. One study described a favorable outcome in children with DS using combined genioglossus advancement (GGA) and radiofrquency ablation (RFA) of the base of the tongue. There were 31 pediatric patients in the study group and 19 were patients with DS. The study showed 58% success rate among patients with DS. The overall improvement in AHI in this study was from 14.1 to 6.4 and increase in the mean nadir oxygen saturation from 87% to 90.9%. Radiofrequency ablation delivers limited thermal damage to the tongue to decrease the bulk and flaccidity of the tongue by fibrosis. In the GGA, the tongue is pulled forward making the tongue firm with less chance of retroglossal collapse during sleep [44]. 

Another tongue-based procedure is midline posterior glossectomy (MPG). MPG and LG were performed in 13 children with DS for treatment of refractory OSA following AT. Patients were evaluated by cine MRI to establish level of obstruction. This study showed that MPG when performed with LT improved the OSA with decrease in AHI from 47 to 5.6 (*p* < 0.05) in normal weight and overweight but not obese children with DS [42]. 

### 4.7. Myofunctional Therapy

Another treatment option that can be used adjunctively in the treatment of OSA is myofunctional therapy. It is also referred to as “oropharyngeal exercises”. Although this therapy is lacking in data, it is used to strengthen the muscles in the upper airway, given that muscular hypotonia is involved in the pathogenesis of OSA in children with DS [37].

Lukowicz et al. examined the efficacy of myofunctional therapy in 42 pediatric patients with DS. These children underwent a one-week intensive training camp that involved three daily sessions of myofunctional exercises that were 45 min each. The average mixed AHI was 6.4 ± 8.6 at baseline, compared to 6.4 ± 10.8 after the treatment (*p* > 0.05). However, the ≤90% desaturation index decreased from 2.7 ± 4.5 at baseline to 2.1 ± 3.7 after treatment (*p* < 0.05) [37]. 

As a result, there was no improvement in OSA severity with one week of intensive myofunctional therapy. More studies with longer treatment duration and follow up are required to better assess the efficacy of this therapy for pediatric patients with DS and OSA.

### 4.8. Pharmacologic Interventions

For pediatric patients with mild OSA or residual disease status post-AT, pharmacological interventions have been used. These include options such as intranasal corticosteroids and oral leukotriene inhibitors to reduce airway inflammation [36]. 

Howard et al. reviewed pediatric DS patients with mild OSA and the efficacy of medication versus observation. They looked at 23 patients under the age of 18 with a baseline AHI between 1 and 5 on PSG. Of those, 10 patients received pharmacologic intervention, with 8 receiving montelukast and 2 receiving fluticasone. There were 13 patients who did not receive any pharmaceutical intervention [36]. Both the pharmaceutical intervention group and the observation group had a median follow-up time of 15.4 months to obtain a follow-up PSG. In the medication arm, the baseline AHI was 3.5 compared to 3.6 on subsequent PSG (*p* = 0.21). They found that in the observation group, the baseline AHI was 2.9 compared to 3.6 on follow-up PSG (*p* = 0.60) [36]. At follow up, three patients in the medication group reported an improvement in nocturnal symptoms, four reported no change, and one reported worsening of symptoms. In the observation group, four patients reported an improvement, two reported no change, and two reported the worsening of nocturnal symptoms [36]. 

A retrospective chart review by Yu et al. examined pediatric patients with DS and mild OSA (obstructive AHI ≤ 5 events/h) who were treated with intranasal corticosteroids and/or montelukast and compared them to patients who underwent simple observation for at least three months. For the 29 children in the group that received pharmacologic intervention, the median time from baseline to follow-up PSG was approximately 14 months. The observation group included 16 children, and their median time from baseline to follow-up PSG was approximately 10.5 months [35]. This study also showed that there were no statistically significant changes in both the medication group (AHI 3.5 at baseline to 3.8 at follow up, with a *p*-value of 0.14) and the observation group (AHI 3.5 at baseline to 4.3 at follow up, with a *p*-value of 0.14) [35]. 

Therefore, the utility of anti-inflammatory medications for OSA in pediatric patients with DS has not yet been well studied. Further studies analyzing the efficacy of these interventions in a larger sample size of this specific population is required, especially as the baseline AHI in these studies is quite low, thereby decreasing the room for significant improvement after the interventions. 

### 4.9. Weight Loss

Obesity is commonly seen in the DS population and can contribute to increased upper airway resistance. Because of the known association between OSA and obesity, weight loss is recommended as part of the therapy for obese children with OSA. Some strategies for weight loss include a balanced diet without energy restriction, vitamin and mineral supplementation, and increased physical activity [51]. A systematic review did show that in pediatric patients aged 10–19, weight loss interventions led to statistically significant reductions in AHI and improved oxygen saturation [52]. However, this study excluded children with DS. In the population of children with DS, there is a lack in literature that analyze whether weight loss intervention results in sustained decreases in AHI or improved OSA symptoms. Though in one study done on children with DS, they did show that on PSG, the oxygen saturation nadir was significantly lower for obese children compared to nonobese children with DS. This study also noted that weight was the most significant risk factor for children with DS, with those who were obese being three times as likely to have severe OSA [53]. This highlights the importance of monitoring weight and continuing to counsel families of children with DS about weight loss.

## 5. Discussion

If pediatric patients with DS are found to have OSA on polysomnography, the first-line treatment option is often AT for patients with enlarged tonsils and adenoid. With AT alone, a 51% reduction in pre-operative AHI can be expected [54]. Although effective in some patients, this procedure is not without the risk of morbidity and mortality, and AT alone may not cure OSA in children with DS. A recent study suggests that as many as 31.5% of patients with DS had postoperative complications following AT, with respiratory issues topping the list of most common problems. These children often possess preexisting conditions associated with DS, such as aerodigestive comorbidities, that appear to put them at increased risk for these respiratory complications following AT. Complications include desaturations, aspiration pneumonia, and prolonged intubation [55]. CPAP therapy and other adjuvant surgical procedures such as lingual tonsillectomy, tongue-based procedures, and hypoglossal nerve stimulation may need to be considered for persistent OSA after AT. In these cases of refractory OSA after AT, drug-induced sleep endoscopy (DISE) has been utilized to evaluate for other potential airway obstructions and has been associated with improvement of subjective and objective measures of sleep. In a small study of 26 children with OSA who completed DISE-directed operative management of the level(s) of ongoing upper airway obstruction, 14 of whom had diagnosed DS, the mean AHI dropped from 7.0 to 3.6 [56]. 

After AT, CPAP is often the most common therapy utilized for treatment in children with OSA. Though data on CPAP therapy does show promising reduction of AHI, recent research in children with DS is few and far between. Though one of the studies did not specifically address DS, it was a pediatric study of 56 children that looked at CPAP vs. Bilevel positive airway pressure (BIPAP) therapy and found no significant differences in AHI. The study does not point out a clear advantage of using BIPAP over CPAP for children with OSA if there is no hypoventilation or central apnea [57]. Children with DS tend to have hypoventilation out of proportion to the severity of their OSA which may make them good candidate for BIPAP when CPAP is not adequate. With autism spectrum disorder commonly being a comorbidity of children with DS [58], sensory issues may call for further techniques of acclimatation and desensitization of mask use before long-term adherence can be possible. In a study measuring the effectiveness of CPAP therapy in adults with DS, adherence was a significant issue [59]. Though programs such as the Sleep Apnea Self-Management Program (SASMP) have been shown to improve the consistency of CPAP use in the general population [60], no such cognitive behavioral interventions have been studied in children with Down syndrome. Given the relatively low complications from positive airway pressure therapy [61], this area would benefit from more robust data regarding not only AHI changes but also strategies to better maximize long-term adherence with CPAP therapy in children with DS. 

An orthodontic treatment that widens the palate and nasal passages known as rapid maxillary expansion or rapid palate expansion also exists for use in the pediatric population, but data for this specific treatment lag behind other therapies, especially in children with DS. A small study looked at 10 children with OSA without diagnosed DS who were followed up years after rapid maxillary expansion treatment, and it showed a decrease in both the AHI and clinical symptoms [62].

Other treatment considerations for OSA in children with DS include turbinate reduction, uvulopalatopharyngoplasty (UPPP), expansion and lateral pharyngoplasty, supraglottoplasty for patients with laryngomalacia and mandibular distraction osteogenesis. There is limited data for these interventions in children, including in those with DS and OSA. 

If AT, CPAP therapy, or any of the other available options fail to control the child’s OSA, some physicians may use tracheostomy as a last resort. Tracheostomy may be indicated in children with DS who have severe OSA with significant sequelae and who did not respond to conventional treatment and are poor surgical candidates. Though effective, complications may include tracheoesophageal fistula, decannulation, and tracheostomy tube obstruction [63].

Given the increased incidence of OSA in children with DS, there is still limited literature on the treatment options and its outcomes. With the increased prevalence of OSA in children with DS and the significant sequelae secondary to untreated OSA, more robust trials on treatment involving children with DS must be performed. Treatment guidelines specific to those with OSA in the DS need to be created.

## Figures and Tables

**Table 1:** Outcomes of interventions in studies of obstructive sleep apnea in pediatric Down syndrome.

Study, Year	N	Age (Years)	Design Method	Intervention	Outcome of Intervention
Caloway et al. (2020) [33]	20	10 to 21	Case series	HGN stimulation	Significant improvement in AHI and OSA-18 scores
Nerfeldt and Sundelin (2020) [34]	14	1 to 17	Retrospective cohort study	AT	Minimal reduction in AHI; significant improvement in OSA-18 scores one year after AT
Yu et al. (2020) [35]	29	Median age: 7.4	Retrospective chart review	Pharmacologic (intranasal corticosteroids and/or montelukast)	Medication therapy was not found to be effective in treating mild OSA
Howard et al. (2020) [36]	23	0 to 10	Retrospective chart review	Pharmacologic (montelukast; fluticasone)	Low rates of resolution of mild OSA
Lukowicz et al. (2019) [37]	42	2 to 11	Prospective longitudinal study	One-week intensive myofunctional therapy training camp	Marginal effect on OSA symptoms
Amaddeo et al. (2019) [38]	6	0 to 17	Prospective study	HFNC	Three of the six participants successfully managed OSA with HFNC
Diercks et al. (2018) [39]	6	10 to 21	Case series	HGN stimulation	Significant improvement in AHI and OSA-18 scores at 6- and 12-month follow ups
Prosser et al. (2017) [40]	21	Mean age: 9.3 ± 4.3	Retrospective case series	LT	Significant improvement in median AHI and mean oxygen saturation nadir
Ingram et al. (2017) [41]	75	0 to 16	Retrospective chart review	Tonsillectomy with concurrent or previous adenoidectomy	Improvement in AHI from baseline 21.3 ± 19.7 to 8.0 ± 8.1 six months after tonsillectomy
Propst et al. (2017) [42]	13	6 to 18	Prospective study	MPG and LT	Improvement in AHI in normal weight or overweight children with DS, but not obese children with DS.
Sudarsan et al. (2014) [43]	124	6 to 12	Prospective, randomized, cohort study	AT or CPAP	Improvement in AHI from baseline 3.83 ± 1.36 to 2.62 ± 0.87 six months after AT Improvement in AHI from baseline 3.46 ± 1.67 to 1.09 ± 0.61 six months after nightly CPAP use
Wooten and Shott (2010) [44]	19	Mean age: 11.5	Retrospective institutional review	GGA and RFA	Significant improvement in mean AHI and mean nadir oxygen saturation
Shete et al. (2010) [45]	11	Mean age: 8.5	Retrospective chart review	AT	Improvement in total AHI from 15.3 to 9.1 No change in REM-AHI, oxygen saturation, distribution in stages of sleep, sleep efficiency, and mean ArI 73% of participants required CPAP, BIPAP, or nocturnal oxygen for persistent OSA

AHI: apnea–hypopnea index; ArI: arousal index; AT: adenotonsillectomy; BIPAP: bi-level positive airway pressure; CPAP: continuous airway pressure; GGA: genioglossus advancement; HFNC: high-flow nasal cannula; HGN: hypoglossal nerve; LT: lingual tonsillectomy; MPG: midline posterior glossectomy; OSA: obstructive sleep apnea; OSA-18: Obstructive Sleep Apnea-18; REM-AHI: rapid eye movement apnea–hypopnea index; RFA: Radiofrequency ablation.
